# A mechanistic review of pharmacological activities of homeopathic medicine licorice against neural diseases

**DOI:** 10.3389/fnins.2023.1148258

**Published:** 2023-03-06

**Authors:** Parvin Zulfugarova, Tayebeh Zivari-Ghader, Sevinj Maharramova, Elham Ahmadian, Aziz Eftekhari, Rovshan Khalilov, Vugar Ali Turksoy, Gvozden Rosić, Dragica Selakovic

**Affiliations:** ^1^Department of Zoology and Physiology, Faculty of Biology, Baku State University, Baku, Azerbaijan; ^2^Department of Medicinal Chemistry, Faculty of Pharmacy, Tabriz University of Medical Sciences, Tabriz, Iran; ^3^Department of Pharmaceutical Technology and Management, Azerbaijan Medical University, Baku, Azerbaijan; ^4^Kidney Research Center, Tabriz University of Medical Sciences, Tabriz, Iran; ^5^Department of Biochemistry, Faculty of Science, Ege University, İzmir, Turkey; ^6^Institute of Molecular Biology and Biotechnologies, Ministry of Science and Education Republic of Azerbaijan, Baku, Azerbaijan; ^7^Research Center for Pharmaceutical Nanotechnology, Biomedicine Institute, Tabriz University of Medical Sciences, Tabriz, Iran; ^8^Department of Biophysics and Biochemistry, Baku State University, Baku, Azerbaijan; ^9^Department of Public Health, Faculty of Medicine, Bozok University, Yozgat, Turkey; ^10^Department of Physiology, Faculty of Medical Sciences, University of Kragujevac, Kragujevac, Serbia

**Keywords:** licorice, Parkinson, Alzheimer’s disease, herbal medicine, brain

## Abstract

The use of medicinal plants has grown in popularity in recent decades because, as natural ingredients, they have fewer adverse effects and are more effective than synthetic alternatives. As a small perennial herb, *Glycyrrhiza glabra* L. (Licorice) has been investigated for its therapeutic efficacy against neural disorders mainly ischemic stroke as well as the neurodegenerative diseases such as dementia and Alzheimer’s disease, and Parkinson’s disease which has been attributed to its HMGB inhibitory function, reactive oxygen scavenging and anti-inflammatory activity. The objective of current review is to review the evidence for the pharmacological effects of licorice and its vital active components on neurological disorders and the underlying signaling networks. We reviewed Papers published from 2000.1.1 up to 2 January 2023 in web of science, Google Scholar and PubMed data bases using key words including “Licorice,” “*Glycyrrhiza glabra* L.,” “Glycyrrhizic acid,” “brain,” “neurodegenerative disease,” “Alzheimer’s,” and “Parkinson” were used to search in title/abstracts. Licorice extract and/or its active components can be used safely in therapeutic doses for optimizing the management of a multiple neurodegenerative disorders, and hampering the extent of neural tissue injury and neurologic deficits subsequent to cerebrovascular accidents.

## 1. Introduction

Licorice root is commonly used in the preparation of commercial products for the food industry, tobacco flavoring, and herbal medicine (Fu et al., 2013). Since ancient eras, licorice has been utilized as a medicinal plant for a variety of human diseases, including infections, neural disorders, peptic ulcers, and asthma (Ayeka et al., 2016). Recent investigations have shown many more medicinal properties. Flavonoids [isoliquiritigenin (ISL), liquiritigenin, LQapioside, and liquiritin (LQ)], isoflavonoids [Dehydroglyasperin C (DGC)], and triterpenes [glycyrrhizic acid (GA) and glycyrrhetinic acid monoglucuronide (GM)] are the active components of licorice root. The sugary flavor of licorice is due to GA (Jiao et al., 2013; Ton et al., 2013; Hosseinzadeh and Nassiri-Asl, 2015; Han et al., 2017). Flavonoids derived from licorice have antimicrobial, anti-inflammatory, antioxidant, and antispasmodic attributes (Guo et al., 2016). Besides, DGC has recently been shown to have anti-cancer properties (Shi et al., 2015). Licorice and its constituents have been shown to mediate several signaling pathways involved in acute and chronic neurodegeneration. Ischemic stroke, which causes a burst of infarctions in the center of a hypoperfusion zone, is an acute neurotoxic process. Neurodegenerative diseases like Parkinson’s and Alzheimer’s are examples of chronic neurotoxicity (AD) (Gaur et al., 2014; Abduljawad et al., 2022; Hassan et al., 2022a). Recent studies showed that plant based active ingredients are effective in neurodegenerative disease (Wei et al., 2021; Hassan et al., 2022b; Mahnashi et al., 2022). Both active components and the entire extract of licorice have been shown to have neuroprotective properties (Hopkins, 2008; Dai et al., 2013; Huang et al., 2016). The licorice root contains several active ingredients with biological functions. Using High-performance liquid chromatography techniques, multiple chemical compounds, including flavonoids and triterpene saponins, have been identified (Hopkins, 2007; Zhu et al., 2018; Heidari et al., 2021). Other minor components identified include DGC, glycerol, glycerin, licoflavone, and glycycoumarin (Gao et al., 2016).

**FIGURE 1:**
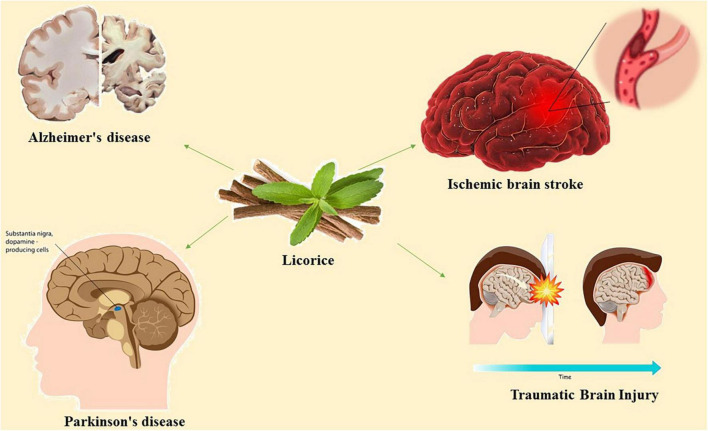
Schematic illustration of the effects of licorice on neural disorders.

Acetylcholinesterase, nitric oxide synthase, cholinesterase, monoamine oxidase A (MAOA), monoamine oxidase B (MAOB), and are among the afferent nervous system targets that licorice influences. Both MAOA and MAOB belong to the monoamine oxidase (MAO) family and play a crucial role in maintaining mental health by catalyzing the oxidative deamination of neurotransmitters and xenobiotic amines (Ramsay and Tipton, 2017). The proper regulation of MAO activity is required for the effective treatment of neurodegenerative diseases. MAO-B inhibition is a well-known treatment strategy for Alzheimer’s disease and Parkinson’s disease (Dezsi and Vecsei, 2017). Various constituents of licorice including licocoumarone, licopyranocoumarin and glycyrrhisoflavone inhibit MAO activity (Hatano et al., 1991; Ramalingam et al., 2018). Most of the inhibitory mechanism of licorice is dependent to the presence of glicoricone and structure of MAO (Hatano et al., 1991). Furthermore, licorice can mediate the function of acetylcholinesterase, a key enzyme in the hydrolysis of acetylcholine (Coloviæ et al., 2013). Licorice contains 52 compounds that have been shown to inhibit acetylcholinesterase activity (Chen et al., 2019). The current review concentrated on the available evidence regarding the pharmacologic effects of active compounds of licorice on neural disorders and the underlying signaling pathways (Figure 1).

## 2. Method of searching

Papers had published from 2000.1.1 up to 2 January 2023 in web of science, Google scholar and pubmed were investigated. 65 papers from web of science database, 73 from Google scholar and 318 in pubmed were find with including criteria (key words) “Licorice.” “*Glycyrrhiza glabra* L.,” “glycyrrhizic acid,” “brain,” “neurodegenerative disease,” “Alzheimer’s,” and “Parkinson” in title/abstracts. Paper without the keywords, review articles, abstracts of congress, and non-English papers were excluded from this review.

## 3. Licorice in ischemic brain stroke

**FIGURE 2:**
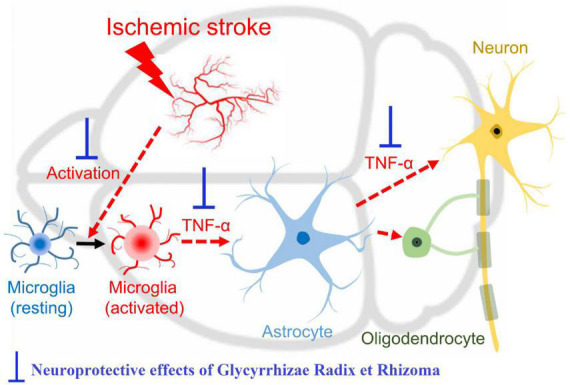
Mechanism of the neuroprotective effect of *Glycyrrhizae Radix et Rhizoma* in the alleviation of inflammation in ischemia/reperfusion-induced brain damage (Choi et al., 2022).

Ischemic stroke is one of the important causes of death worldwide, causing irreversible brain tissue damage. Current ischemic stroke mainstay therapy includes blood supply recovery, however, blood supply reestablishment is not obtained during the golden time due to the patient’s late arrival or contraindications related to the use of endovascular and thrombolytic agents (Roaldsen et al., 2021). Various agents have been proposed to reduce ischemia-related neural tissue injury by inhibiting inflammatory and neurotoxic pathways (DeLong et al., 2022). Licorice-derived glabridin has substantially modulated the middle cerebral artery occlusion (MCAO)- induced cerebral injuries in rats and also in staurosporine-treated cultured rat cortical neurons. The results indicated that glabridin escalated the levels of endogenous antioxidants and prevents cellular apoptosis (Yu et al., 2008). It has been shown that post-treatment of the ischemic stroke mice with 125 mg/kg *Glycyrrhizae Radix et Rhizoma* was effective in cerebral infarction and inflammatory response by regulating the activation of microglia and astrocytes (Figure 2; Choi et al., 2022).

In animal models, licorice effectively blocked neuroexcitatory damage cascades (Wei et al., 2021). It has also significantly reduced lactate dehydrogenase release in hypoxia-induced cultured gerbil hippocampus (Hwang et al., 2006). *In vivo*, licorice treatment has increased superoxide dismutase activity in a carotid artery occlusion model (Sathyamoorthy et al., 2020). In another study, intravenous administration of GA after ischemia induction significantly reduced infarction size, microglia activation, and the production of pro-inflammatory cytokines (Kim et al., 2012). GA in combination with candesartan have significantly ameliorated the expression of toll like receptor (TLR) (TLR-2 and TLR-4) and subsequent downstream inflammatory markers (Barakat et al., 2014). The activity of HMGB is linked to post-ischemia inflammation of neural tissue (Ramalingam et al., 2018). GA, a known HMGB inhibitor, has reduced the inflammatory response in mice with MCAO (Lim et al., 2018). The mechanism of this inhibition has been attributed to the HMGB1-TLR4-IL-17A signaling pathway (Zhang et al., 2014). In another study GA has exerted protective effects on ischemia-reperfusion injury in rat brains through the prohibition of oxidative stress, inflammation, and apoptotic injury by inhibiting the cytokine activity of HMGB (Gong et al., 2014). Also, the HMGB1 inhibitory role of GA has been shown to be connected with ferroptosis and the related signaling network. Ferroptosis is a caspase-independent type of cell death triggered by lipid peroxidation and could be caused as a result of glutathione peroxidase impairment (Wang et al., 2018). GL can prevent neuronal ferroptosis, suppress oxidative stress, diminish mitochondrial injury, and decrease neuro-inflammation in HIBD *via* the HMGB1/GPX4 pathway (Zhu et al., 2022). Furthermore, GA treatment can significantly reduce CD68+ macrophage infiltration, indicating a role in T-cell-mediated cytotoxicity (Xiong et al., 2016). ISL derived from licorice has been shown to reduce the expression of apoptotic factors and the formation of reactive oxygen species (ROS) in neural tissues (Hwang and Chun, 2012). The first clinical trial of licorice extract in dried powder capsules found that it effectively improved neurologic function after the onset of ischemic stroke symptoms (Ravanfar et al., 2016). In this study 450 and 900 mg licorice extract was orally prescribed for 7 days and National institute of Health stroke scale (NIHSS) and Modified Rankin Scale (MRS) scores were evaluated prior to licorice intake and 90 days after treatment.

## 4. Licorice in Alzheimer’s disease

Alzheimer’s disease (AD) is a neurodegenerative condition defined by the gradual death of brain cells through many signaling pathways, including glutamate, PI3K/Akt, extracellular signal-regulated kinase (ERK), HMGB, and Necrotic factor kappa B (NFkB) (Kao et al., 2009; Srinivasan and Lahiri, 2015; Miculas et al., 2022). Studies showed that GA blocked the activity of NF-kB as a key element of neurodegenerative disease pathogenesis (Wang et al., 2011). GA has been shown to inhibit the activity of NFkB, which is essential in the pathogenesis of neurodegenerative diseases (Hwang et al., 2006). Following activation, NFkB sends several downstream signals that terminate in inflammation (Shih et al., 2015). Glutamate has been shown to induce apoptosis in cultured hippocampal cells, which has been confirmed by microscopic analysis of the morphological properties of apoptosis. At the same time, GA treatment may impair apoptotic machinery function in a concentration-dependent manner. In this study, GA significantly reduced glutamate-mediated NMDA receptor signaling and prevented the activation of NFkB as a downstream signal in the mentioned pathway (Cherng et al., 2006). Another study found that GA and GM inhibited NFkB and other inflammatory pathways in an LPS-induced inflammation model (Wang et al., 2011). Furthermore, GA and GM have been shown to lower Bcl-2 levels and increase PI3K signaling activity, resulting in the inhibition of cytotoxic mechanisms. Also, DGC administration has been shown to reduce the inflammatory response to LPS and NFkB activity in microglial cells (Kim et al., 2013). ERK signaling is another important pathway involved in the potential neuroprotective effects of GA. GA has been shown to increase ERK activity in neural cultures (Wang et al., 2014). Licorice-derived ISL inhibits glutamate-related neurotoxicity by decreasing stress mediators such as ROS, membrane lipid peroxidation, calcium influx, decreasing apoptosis signaling markers, and increasing cell survival factors. In addition, by suppressing ROS formation and blocking the release of apoptotic factors (Bcl2, Bax, and AIF) from mitochondria into the cytosol, ISL has attenuated glutamate-induced mitochondrial injury and further hippocampal neural loss (Peng et al., 2015). It has been reported, licorice reduced microglial cell activation and inflammation in LPS-induced neurotoxicity by inhibiting activator protein 1 (AP1) and NFkB. This suppression can prevent neurotoxic processes in inflammatory-related neural disorders such as Alzheimer’s (Zhu et al., 2022). Indeed, ILS has been shown to significantly reduce amyloid peptide (2535) [A(2535)]-induced neurotoxicity by modulating downstream signaling mediators (Ahn et al., 2010; Lee et al., 2012). Interestingly, licorice has been shown to improve cognitive markers of Alzheimer’s disease *in vivo*. Licorice has been shown to have anticholinesterase activity in reversing scopolamine and diazepam-induced amnesia. Anticholinesterase agents are well-known medications used in the treatment of Alzheimer’s disease (Dhingra et al., 2004). Three consecutive recipients of licorice-derived glabridin were able to effectively reduce cholinesterase activity comparable to standard medication (Cui et al., 2008). Glabridin decreases MDA levels in rat brains while raising superoxide dismutase and glutathione levels (Yu et al., 2008). A research demonstrated that feeding hypoxic rats with *G. glabra* restored low levels of brain neurotransmitters such as glutamate and dopamine and decreased AChE activity.

Another study tested the neuroprotective properties of an aqueous root extract of *G. glabra* in Wistar albino rats. The dosages of 150 and 225 mg/kg showed a considerable neuroprotective effect. The neuroprotective action is linked to the presence of the active isoflavone “Glabridin” in *G. glabra* (Hasanein, 2011). Furthermore, when used for 30 days, higher concentrations reversed diabetes-induced memory and learning dysfunction *in vivo* (Hasanein, 2011).

Recent research has found that HMGB1 plays a pathogenic role in memory impairment, primarily *via* the TLR4 and RAGE signaling pathways (Rong et al., 2021; Miculas et al., 2022). Furthermore, HMGB1 neutralization has been shown to reduce cognitive dysfunction and post-TBI cognitive impairment (Hei et al., 2018; Okuma et al., 2019). TLR4 and NF-B phosphorylation, followed by activation of the NLRP3 inflammasome, is one proposed mechanism by which HMGB1 affects cognition (Costello et al., 2011). Previous research has found that NLRP3 contributes to the worsening of cognitive dysfunction (Li et al., 2017). In LPS-treated animal models, GA has been shown to slow memory decline (Song et al., 2013). GA protects by lowering the expression of inflammatory markers such as TNF- and IL-1, as well as the protein expression of COX-2 and iNOS (Song et al., 2013). In addition, by inhibiting HMGB1/NF-B signaling-mediated neuroinflammation, GA treatment improved spatial memory in isoflurane-exposed animals (Wang et al., 2016). By preventing brain inflammation and AD-like pathology through HMGB1 neutralization, GA has been found to protect mice from surgery-induced cognitive impairments (short swimming latency and distance in the MWM test) (Kong et al., 2017). GA also can significantly decrease inflammatory markers, NF-B, and hippocampal A levels (Kong et al., 2017). GA has been shown to reduce cell death in AD experimental models by inhibiting HMGB1 (Jang et al., 2013).

## 5. Licorice in Parkinson’s disease

Another significant neurodegenerative disorder is Parkinson’s disease, which is characterized by neural loss and gliosis in the substantia nigra. In Hwang and Chun (2012), the first study using licorice to treat Parkinson’s disease was conducted, in which 6hydroxydopamine (6OHDA)-induced neurotoxicity was used to mimic PD-like dysfunction in dopaminergic neurons *in vivo*. It was discovered that ISL, by mediating intracellular signals, could significantly reduce ROS formation and inhibit the release of apoptotic factors. ISL and liquiritigenin have been shown to significantly reduce synuclein fibril deposition (the pathologic hallmark of Parkinson’s disease) in neural tissues. Furthermore, ISL has the potential to disaggregate previously formed deposits (Liao et al., 2016).

The pathophysiology of Parkinson’s disease is linked to several signaling axes that are involved in cell survival, protein aggregation, inflammation, oxidative stress, apoptosis, mitochondrial damage, and autophagy (Angelopoulou et al., 2019; Paudel et al., 2020). The aggregation of -synuclein-containing Lewy bodies causes cognitive and motor dysfunction (Angelopoulou et al., 2018; Kirkeby and Barker, 2019). Furthermore, HMGB1 signaling appears to be tightly linked with inflammatory response and degeneration in Parkinson’s disease, as increased levels of HMGB1 have been detected in PD patients (Yang et al., 2018; Baran et al., 2019). Therefore, HMGB1 targeting has great potential as a treatment for PD (Song et al., 2013; Wang et al., 2016). Lower levels of HMGB1 and RAGE in the midbrains of MPTP-treated rats were associated with this protective effect (Kong et al., 2017). Increasing antioxidant protein levels and lowering MDA and carbonyl production, another research found that GA and 18-glycyrrhetinic acid (a metabolite of GA) prevented cell death in differentiated PC12 cells treated with MPTP and 1-methyl-4-phenylpyridinium (MPP+) (Kim and Lee, 2008). Furthermore, the combination of GA and 18-glycyrrhetinic acid has been shown to improve caspase 3 activity GA and 18-glycyrrhetinic acid was found to inhibit mitochondrial permeability transition in MPP+-induced neurotoxicity (Yim et al., 2007). GA has also been shown to have neuroprotective effects in the rotenone-induced Parkinson’s disease model by increasing intracellular glutathione levels, decreasing MDA, increasing cellular antioxidant capacity, and decreasing pro-inflammatory cytokine release (Ojha et al., 2016).

**FIGURE 3:**
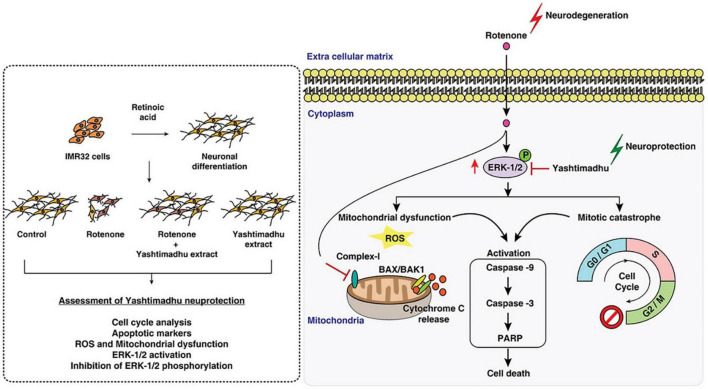
Mechanism of licorice’s neuroprotective action against rotenone-induced toxicity. Reproduction authorized. Elsevier^©^ 2021 Elsevier B.V. (Karthikkeyan et al., 2021).

Rotenone induces Parkinson’s disease-associated cell cycle re-entry-mediated G2/M arrest, mitochondria-related oxidative stress, and triggering of the caspase-3 apoptotic pathway through MEK-ERK-1/2 hyperactivation (Karthikkeyan et al., 2021). Glycyrrhiza glabra L, when used in combination with other therapies, has been shown to decrease cellular ROS and improve mitochondrial health (Karthikkeyan et al., 2021). By downregulating the MEK-ERK-1/2 axis, it stops the cell cycle from restarting after a mitotic catastrophe and stops caspase activation. Findings suggest that *G. glabra* L protects cells against neurotoxic stress (Figure 3; Karthikkeyan et al., 2021).

These results indicate that licorice and its compounds may possess neuroprotective capabilities against Parkinson’s disease. However, further study is required to determine the therapeutic effectiveness and safety of different formulations.

## 6. Licorice in traumatic brain injury

Traumatic Brain Injury (TBI) is a brain injury caused by an external mechanical force, such as a blow to the head (Webster et al., 2017). There are two types of traumatic brain injury depending on the underlying mechanism: closed and penetrating. The severity of a traumatic brain injury is measured by structural damage and the Glasgow coma scale (GCS) (Maas et al., 2008). TBI encompasses both primary and secondary damage. The primary damage consists of an external force disrupting the blood-brain barrier (BBB), which is followed by an increase in inflammatory markers, ROS formation, excitotoxicity, and neural cell death (Woodcock and Morganti-Kossmann, 2013; Parker et al., 2017). Intracranial pressure (ICP), seizures, infection, and hematomas are all caused by the inflammatory response. The secondary injury occurs next, which can be reversed to improve (Parker et al., 2017). Untreated brain injuries can cause behavioral and cognitive disruption, seizures, chronic encephalopathy, and Alzheimer’s disease (Barman et al., 2016; Hay et al., 2016). Despite recent advances in basic and clinical research, treatment options with favorable outcomes following TBI remain limited (Hasanein, 2011). Though, numerous strategies have been proposed for TBI treatment, with inflammation targeting by pharmaceutical agents being a particularly prominent option (Kochanek et al., 2015). Necrotic neurons release HMGB1 during TBI *via* an N-methyl D-aspartate receptor subtype 2B (NR2B)-mediated mechanism (Richard et al., 2017). When HMGB1 is produced, it binds to TLR2, TLR4, and RAGE, initiating the HMGB1/TLR4/RAGE/NF-B cascade, which leads to the release of inflammatory cytokines and further aggregation of the secondary damage (Yang et al., 2005; Gu et al., 2014). GA has been shown to have promising results in animal models of TBI. For example, in the fluid percussion injury (FPI)-induced model of TBI, GA could, in a concentration-dependent manner, block the translocation of HMGB1 from the nucleus to the cytosol and thus protect BBB permeability (Cai et al., 2016). Furthermore, GA has been shown to improve cognitive function and locomotor activity (Parker et al., 2017). Another pre-clinical study found that GA treatment improved walking balance while decreasing brain edema and apoptosis (Gu et al., 2014). GA significantly lowered cytoplasmic expression of HMGB1 and the number of TLR4 and RAGE positive cells. GA’s neuroprotective benefits were ascribed mostly to its anti-inflammatory action through HMGB1 inhibition (Figure 3).

The pre-treatment of C57Bl/6 mice with GA before the imitation of TBI had a significant impact on the reduction of HMGB1 levels in the brain. However, administering GA 1 h after TBI did not produce the same results, whereas chronic use of GA may improve memory and spatial learning. GA administration in TBI-induced animals may also mediate the polarization of microglia associated with secondary injury (Gao et al., 2018). In a focal contusion animal model, GA has been shown to reduce neurological function recovery, lesion volume, and HMGB1 expression. Notably, GA inhibited post-TBI M1 phenotype activation, increased M2 phenotype activation, and reduced TBI consequences, most likely by blocking an M1-like pro-inflammatory phenotype in microglia and, in part, inhibiting HMGB1 (Gao et al., 2018). These findings suggest that targeting HMGB1 to mediate microglia polarization could be a promising therapeutic option for TBI.

**FIGURE 4:**
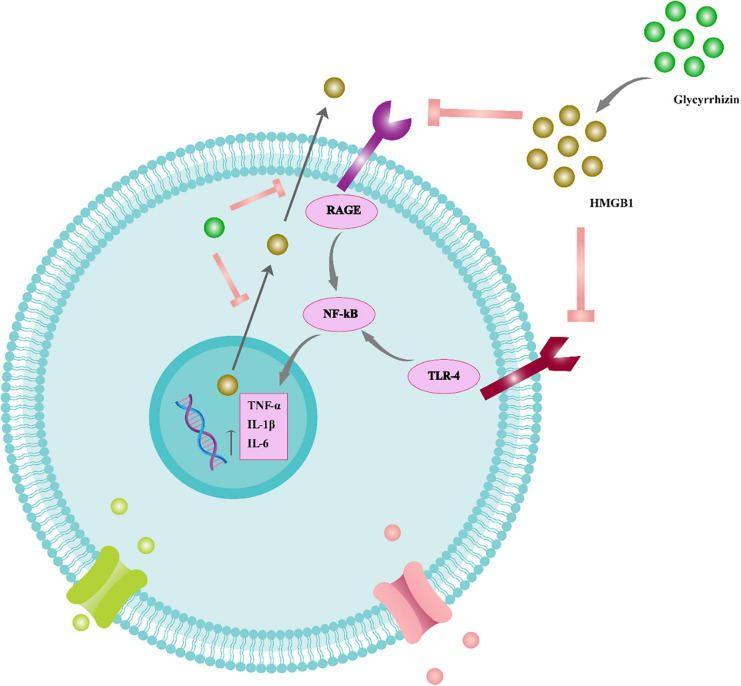
The function of glycyrrhizin in the inflammatory cascade of the neural system. Glycyrrhizin binds to HMGB1 and prevents HMGB1/RAGE and HMGB1/TLR4 interaction. Therefore, NF-B signaling is suppressed and pro-inflammatory cytokines such as TNF-, IL-6, and IL-1 are reduced. Furthermore, glycyrrhizin prevents nuclear translocation of HMGB1 to the cytoplasm and consequent extracellular release, thus also reducing HMGB1’s extracellular pro-inflammatory actions. Tumor necrosis factor (TNF), receptor for advanced glycation end products (RAGE), interleukin (IL), nuclear factor light chain enhancer of activated B cells (NF-B), toll-like receptor 4 (TLR4).

Glycyrrhizic acid treatment has also been shown to suppress apoptosis, reduce axonal damage, inhibit the release of pro-inflammatory cytokines, and improve cognitive impairments in patients with diffuse axonal injury (Pang et al., 2016). As a result, GA treatment may be an effective therapy for various brain injuries. However, the precise underlying mechanisms of neuroprotection must be determined (Figure 4).

The pre-treatment of C57Bl/6 mice with GA before the imitation of TBI had a significant impact on the reduction of HMGB1 levels in the brain. However, administering GA 1 h after TBI did not produce the same results, whereas chronic use of GA may improve memory and spatial learning. GA administration in TBI-induced animals may also mediate the polarization of microglia associated with secondary injury (Gao et al., 2018). In a focal contusion animal model, GA has been shown to reduce neurological function recovery, lesion volume, and HMGB1 expression. Notably, GA inhibited post-TBI M1 phenotype activation, increased M2 phenotype activation, and reduced TBI consequences, most likely by blocking an M1-like pro-inflammatory phenotype in microglia and, in part, inhibiting HMGB1 (Gao et al., 2018). These findings suggest that targeting HMGB1 to mediate microglia polarization could be a promising therapeutic option for TBI.

**FIGURE 5:**
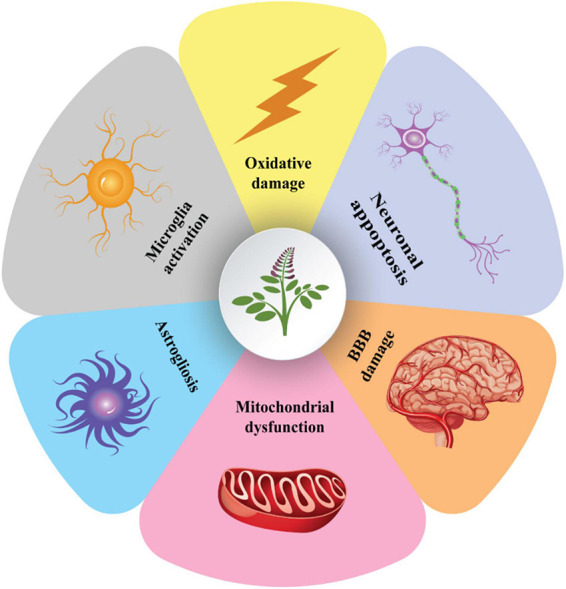
The cellular processes behind the neuroprotective properties of glycyrrhizin. Glycyrrhizin may enhance the integrity of the blood-brain barrier by preventing astrogliosis, neuronal apoptosis, microglia activation, oxidative-induced cellular damage, and mitochondrial dysfunction. These methods enable glycyrrhizin to boost anti-excitotoxicity (for epilepsy treatment), decreasing axonal damage and brain edema (TBI improvement), cognitive and motor function improving (AD and PD treatment), and reducing demyelination (MS treatment). MS, multiple sclerosis; TBI, traumatic brain injury; PD, Parkinson; AD, Alzheimer’s disease.

Glycyrrhizic acid treatment has also been shown to suppress apoptosis, reduce axonal damage, inhibit the release of pro-inflammatory cytokines, and improve cognitive impairments in patients with diffuse axonal injury (Pang et al., 2016). As a result, GA treatment may be an effective therapy for various brain injuries. However, the precise underlying mechanisms of neuroprotection must be determined (Figure 5).

## 7. Possible toxicity of licorice

Some worries have been expressed concerning prolonged high-dosage ingestion of licorice and its compounds, notwithstanding the apparent therapeutic potential. A large amount of licorice may have adverse consequences, and GA and GM are to blame. Renal 11-hydroxysteroid dehydrogenase2, an enzyme involved in the breakdown of cortisol, is inhibited by GA. Forbidden foods may trigger hypermineralocorticoid states, which in turn can lead to moderate hypertension because of elevated potassium and sodium/water retention excretion. Safe human dosing ranges for GA are between 0.015 and 0.229 mg/kg body weight per day (Isbrucker and Burdock, 2006; Asl and Hosseinzadeh, 2008).

It should be noted that different licorice preparations contain varying levels of GA and glycyrrhizin. As a result, the precise concentration of the manufactured preparations should be measured to adjust the dosage within safe limits. The toxicological test results show that licorice has no carcinogenic and/or teratogenic effect *in vivo*. In addition, therapeutic doses of licorice are considered safe for humans based on toxicological assay recommendations and without developmental or reproductive harm (Cosmetic Ingredient Review Expert Panel, 2007). Doses of 900 mg whole extract three times per day for 1 week did not affect human blood pressure or electrolyte hemostasis (Ravanfar et al., 2016). For an additional 8 weeks, healthy volunteers were given 0, 1, 2, and 4 mg/kg/day doses of GA. A six-gr daily licorice intake for a 60 kg individual was found to have no known side effects (van Gelderen et al., 2000).

## 8. Future perspective

Currently the treatment of certain neural disorders is not possible. For instance, medications for ischemic stroke should be prescribed within a short duration after the ischemic attack. On the other hand, current therapeutic option for specific molecular targeting of neurodegenerative disorders are few and costly. Recent studies have been devoted on enlightening novel pharmacologic specifications of the well-known herbal remedy, licorice extract, and its active constituents such as GA, GL, ISL, and glabridin. The newly discovered neuroprotective effects of licorice has provided a new shift in paradigm of neural disease treatment plausible for both acute and chronic brain damages. The active substances of licorice can effectively inhibit cytotoxic pathways in brain. Whole licorice extract and/or purified ingredient can hamper the volume of infarction after ischemic injuries *in vivo*. HMGB has been revealed to be one of the major cellular pathways in the neuroprotective effects of licorice. Combining separated phytochemical elements from licorice and their biological significance in battling multiple neurological disorders and their secondary metabolites may lead to the creation of potential pharmacological formulations.

## 9. Conclusion

To summarize the present review, licorice extracts and flavonoids have been employed to reduce neuro-inflammatory processes after acute ischemia injury to brain cells, TBI, and neurodegenerative diseases. Licorice is safe for human intake at therapeutic doses that have been researched. These results can lead to the discovery and manufacture of novel medications for neurodegenerative illnesses and acute brain tissue injury. However, further ***in vivo*** and clinical studies are needed to extrapolate their action method into other neuro-therapeutic actions.

## Author contributions

PZ: data curation, formal analysis, investigation, and writing—original draft. TZ-G and SM: methodology, visualization, and formal analysis. EA: formal analysis, methodology, and visualization. VT: methodology, visualization, formal analysis, data curation, and writing—review and editing. DS and GR: methodology, formal analysis, data curation, writing—original draft, and writing—review and editing. RK: resources, writing—original draft, and writing—review and editing. AE: conceptualization, study design, supervision, manuscript revision, and final approval of the version to be declaration of competing interest. All authors contributed to the article and approved the submitted version.
